# Kinesin-5 Is Dispensable for Bipolar Spindle Formation and Elongation in Candida albicans, but Simultaneous Loss of Kinesin-14 Activity Is Lethal

**DOI:** 10.1128/mSphere.00610-19

**Published:** 2019-11-13

**Authors:** Irsa Shoukat, Corey Frazer, John S. Allingham

**Affiliations:** a Department of Biomedical and Molecular Sciences, Queen’s University, Kingston, Ontario, Canada; b Department of Molecular Microbiology and Immunology, Brown University, Providence, Rhode Island, USA; Carnegie Mellon University

**Keywords:** *Candida albicans*, yeast, kinesin, microtubule dynamics, mitotic spindle

## Abstract

Mitotic spindles assume a bipolar architecture through the concerted actions of microtubules, motors, and cross-linking proteins. In most eukaryotes, kinesin-5 motors are essential to this process, and cells will fail to form a bipolar spindle without kinesin-5 activity. Remarkably, inactivation of kinesin-14 motors can rescue this kinesin-5 deficiency by reestablishing the balance of antagonistic forces needed to drive spindle pole separation and spindle assembly. We show that the yeast form of the opportunistic fungus Candida albicans assembles bipolar spindles in the absence of its sole kinesin-5, *Ca*Kip1, even though this motor exhibits stereotypical cell-cycle-dependent localization patterns within the mitotic spindle. However, cells lacking *Ca*Kip1 function have shorter metaphase spindles and longer and more numerous astral microtubules. They also show defective hyphal development. Interestingly, a small population of *Ca*Kip1-deficient spindles break apart and reform two bipolar spindles in a single nucleus. These spindles then separate, dividing the nucleus, and then elongate simultaneously in the mother and bud or across the bud neck, resulting in multinucleate cells. These data suggest that kinesin-5-independent mechanisms drive assembly and elongation of the mitotic spindle in C. albicans and that *Ca*Kip1 is important for bipolar spindle integrity. We also found that simultaneous loss of kinesin-5 and kinesin-14 (*Ca*Kar3Cik1) activity is lethal. This implies a divergence from the antagonistic force paradigm that has been ascribed to these motors, which could be linked to the high mitotic error rate that C. albicans experiences and often exploits as a generator of diversity.

**IMPORTANCE** Candida albicans is one of the most prevalent fungal pathogens of humans and can infect a broad range of niches within its host. This organism frequently acquires resistance to antifungal agents through rapid generation of genetic diversity, with aneuploidy serving as a particularly important adaptive mechanism. This paper describes an investigation of the sole kinesin-5 in C. albicans, which is a major regulator of chromosome segregation. Contrary to other eukaryotes studied thus far, C. albicans does not require kinesin-5 function for bipolar spindle assembly or spindle elongation. Rather, this motor protein associates with the spindle throughout mitosis to maintain spindle integrity. Furthermore, kinesin-5 loss is synthetically lethal with loss of kinesin-14—canonically an opposing force producer to kinesin-5 in spindle assembly and anaphase. These results suggest a significant evolutionary rewiring of microtubule motor functions in the C. albicans mitotic spindle, which may have implications in the genetic instability of this pathogen.

## Abstract (precis)

Candida albicans is one of the most prevalent fungal pathogens of humans and can infect a broad range of niches within its host. This organism frequently acquires resistance to antifungal agents through rapid generation of genetic diversity, with aneuploidy serving as a particularly important adaptive mechanism. This paper describes an investigation of the sole kinesin-5 in C. albicans, which is a major regulator of chromosome segregation. Contrary to other eukaryotes studied thus far, C. albicans does not require kinesin-5 function for bipolar spindle assembly or spindle elongation. Rather, this motor protein associates with the spindle throughout mitosis to maintain spindle integrity. Furthermore, kinesin-5 loss is synthetically lethal with loss of kinesin-14—canonically an opposing force producer to kinesin-5 in spindle assembly and anaphase. These results suggest a significant evolutionary rewiring of microtubule motor functions in the C. albicans mitotic spindle, which may have implications in the genetic instability of this pathogen.

## INTRODUCTION

The mitotic spindle is a highly dynamic microtubule (MT)-based structure that undergoes a distinct set of morphological changes in order to correctly attach, orient, and then separate sister chromatids in the dividing cell. Kinesin motor proteins play major roles in shaping and organizing MTs within the spindle over the course of cell division. Early in mitosis, evolutionarily conserved kinesin-5 proteins cross-link the overlapping plus ends of interpolar MTs from newly duplicated centrosomes (spindle pole bodies in yeast) and then slide them apart via plus-end-directed motility to establish spindle bipolarity (1–8). Genetic or chemical inhibition of kinesin-5 activity produces monopolar spindles or inward collapse of preanaphase spindles, usually leading to cell death (9–11). This spindle defect arises from loss of outward forces needed to counterbalance the inward forces supplied by MT minus-end-directed kinesin-14 motors, which pull spindle poles together (10, 12, 13). In many organisms, a nearly normal spindle phenotype can be restored by inactivating or depleting cells of kinesin-5 and kinesin-14 simultaneously because this force imbalance is eliminated (10, 13–16). In this experimental scenario, pushing forces generated by MT growth are sufficient to promote spindle pole separation and bipolar spindle assembly (17–20).

This interplay of motor and MT forces has been studied extensively in the model yeasts Saccharomyces cerevisiae and Schizosaccharomyces pombe (10, 17, 18, 20–22). S. cerevisiae encodes two kinesin-5 homologs, Kip1 and Cin8, that have overlapping, but nonequivalent functions during mitosis (1, 2), while S. pombe encodes a single kinesin-5, named Cut7 (23). All three of these proteins form homotetramers that exhibit bidirectional motility, and all of them function in bipolar spindle assembly and cross-link parallel MTs to help focus kinetochore clusters (24–29). They are also important for stabilizing the overlapping array of MTs at the anaphase spindle midzone and for promoting and regulating timely anaphase spindle elongation (17, 22, 30–34). In both yeast species, loss or inhibition of kinesin-5 function is lethal. However, simultaneous inactivation of their kinesin-14 motors (Kar3Cik1 and Kar3Vik1 in S. cerevisiae or Pkl1 and Klp2 in S. pombe) neutralizes kinesin-5 deficiency (35–38), highlighting the importance of keeping inward and outward forces acting on the spindle in balance. In contrast to the lethality of kinesin-5 loss, bipolar spindles are able to form in the absence of kinesin-14 activity, but are either short and disorganized, or their MT minus ends are unfocused and extend past the opposite spindle pole (39, 40). Our studies of the homologous motors in the opportunistic fungus Candida albicans indicate that these phenotypes, and the opposing relationship of kinesin-5 and kinesin-14 proteins in spindle regulation, are not as highly conserved among eukaryotes as previously thought.

C. albicans is a close relative of S. cerevisiae and S. pombe, but it encodes only one kinesin-5 and one kinesin-14 motor, named *Ca*Kip1 and *Ca*Kar3, respectively, the latter of which forms a heterodimer with a noncatalytic kinesin-like protein, *Ca*Cik1 (41). C. albicans is viable without *Ca*Kip1 (42), and cells lacking *Ca*Kar3Cik1 activity often arrest with a monopolar spindle or two dissociated half-spindles (41). Through further investigation of these unconventional phenotypes, we found that *Ca*Kip1 is not needed for bipolar spindle assembly or nuclear division, even though it exhibits the same cell-cycle-dependent localization as its homologs in budding yeast. However, *kip1*Δ/Δ spindles are shorter and intermittently disassemble prior to cell division. When spindle disassembly occurs, two or more independent bipolar spindles emerge that either segregate between the mother and daughter cells or elongate across the bud neck. Each bring portions of the nucleus with them, which are further subdivided when the spindles undergo anaphase. Rather than neutralizing these kinesin-5 deficiencies, we found that simultaneous loss of kinesin-14 activity is lethal. These results imply that C. albicans Kip1 and Kar3Cik1 have mostly overlapping rather than antagonistic functions in bipolar spindle assembly and that their combined loss cannot be compensated for by MT polymerization forces or other spindle-associated factors.

## RESULTS

### Localization of C. albicans Kip1 mirrors other yeast kinesin-5s.

Like many mitotic proteins, the localization and function of kinesin-5 motors changes throughout the cell cycle. During spindle assembly, S. cerevisiae Kip1 and Cin8, as well as S. pombe Cut7, are enriched at the minus ends of nuclear MTs, toward the spindle poles (26, 43, 44). Here, they are thought to capture MTs emanating from neighboring spindle pole bodies (SPBs) to establish antiparallel MT interactions and provide outward sliding forces to support SPB separation (7, 23–27, 29, 45). Persistence of kinesin-5 near spindle poles in metaphase has been attributed to their interaction with kinetochores, or to kinetochore MTs (kMTs), where they could cross-link parallel kMTs and regulate their assembly dynamics to help achieve chromosome congression (28, 46, 47). Upon anaphase onset, kinesin-5 motors relocate toward the plus ends of interpolar microtubules (ipMTs), which overlap in an antiparallel array in the spindle midzone. Here, their MT cross-linking and plus-end-directed motility help stabilize and elongate the spindle, fully separating the two opposing SPBs, leading to final chromosome segregation (17, 22, 30–34). Recent studies suggest that this cell-cycle-dependent redistribution of yeast kinesin-5s in the spindle is enabled by their capacity for bidirectional motility (24–27, 48, 49).

**FIG 1:**
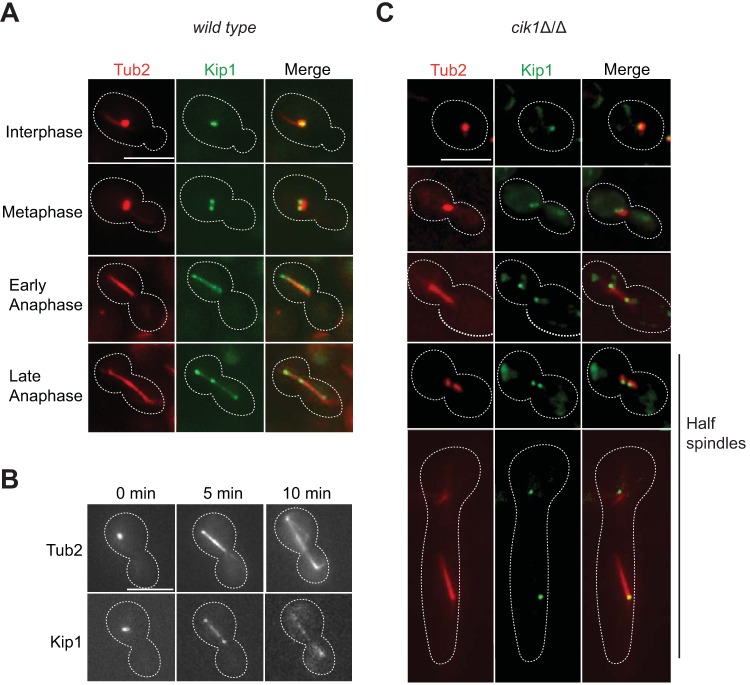
*Ca*Kip1 exhibits similar localization to other kinesin-5s during the cell cycle. (A) Images of wild-type cells expressing Tub2-mCherry and Kip1-GFP (strain CF338). Representative cells from different stages of mitosis were selected. (B) Individual frames from time-lapse microscopy of cells expressing GAL-Tub2-mNeon and Kip1-mScarlet (strain CF443). (C) Images of *cik1*Δ/Δ cells expressing GAL1-Tub2-mCherry and Kip1-GFP (CF340). All cells were obtained from logarithmically growing, unsynchronized cultures in SDC-sucrose medium at 30°C. Scale bars, 5 μm.

The discovery that C. albicans is viable without *Ca*Kip1 (42) suggests that its localization and/or function may be different from those of other yeast kinesin-5s. However, when we imaged fields of unsynchronized cells expressing green fluorescent protein (GFP)-labeled *Ca*Kip1 and mCherry-labeled tubulin (Tub2), we observed similar cell-cycle-dependent motor localization patterns within the mitotic spindle as seen in other yeasts. In small-budded early mitotic cells, *Ca*Kip1 localized near one end of monopolar spindles (in which SPBs are adjacent) (Fig. 1A, row 1) and was found at both poles after SPB separation and bipolar spindle assembly (Fig. 1A, row 2). In cells that were entering anaphase, *Ca*Kip1-GFP fluorescence was dispersed along the length of the spindle. In late anaphase cells, *Ca*Kip1 accumulated at the spindle midzone. The same localization patterns were seen when we imaged individual cells expressing *Ca*Kip1-mScarlet and Tub2-Neon over the course of mitosis by time-lapse microscopy, although photobleaching affected the ability to detect *Ca*Kip1 at later time points (Fig. 1B). To determine whether midzone clustering of *Ca*Kip1 requires overlapping arrays of antiparallel ipMTs in this region, we imaged *Ca*Kip1-GFP in fields of unsynchronized cells lacking kinesin-14 activity (*cik1*Δ/Δ). In other yeasts, kinesin-14 is important for organizing antiparallel ipMT interactions in the midzone so that kinesin-5 motors can properly cross-link and slide antiparallel spindle MTs (39, 40, 50). Without kinesin-14 activity, *Ca*Kip1 remains exclusively near the poles of bipolar spindles and one pole of dissociated half-spindles, presumably due to paucity of antiparallel ipMT overlaps (Fig. 1C).

### C. albicans forms bipolar spindles without kinesin-5.

To understand the role of *Ca*Kip1 in mitosis, we used PCR- and CRISPR-based gene targeting to generate two independent homozygous *CaKIP1* deletion strains. Wary that *Ca*Kip1 could be essential for cell growth (51), we also engineered a conditional *CaKIP1* gene expression strain using the tetracycline-regulatable (TR) promoter system, which enables tight repression of *CaKIP1* in the presence of doxycycline (DOX) (52, 53). Transformants of each strain were screened by PCR to confirm the intended gene modification (data not shown). We further used transcriptome sequencing (RNA-seq) analysis to confirm absence of *CaKIP1* expression in the gene deletion strains (see Table S1 in the supplemental material). The RNA-seq data showed that there were no changes in expression of any other molecular motors or MT-associated proteins (MAPs) to suggest the presence of compensatory mechanisms from such proteins.

10.1128/mSphere.00610-19.1 TABLE S1 (Tab 1) Annotated list of genes that were up- or downregulated more than 2-fold in different *kip1*Δ/Δ strains compared to the wild type. Values are given as log_2_ fold changes. (Tab 2) Data from tab 1 are clustered into Gene Ontology categories for genes important in cell cycle and growth. (Tab 3) Gene expression comparison for motors and motor-associated proteins in response to Ca*KIP1* deletion. There were no significant changes detected in the expression of other motor and motor-associated proteins in the absence of Kip1. Download Table S1, XLSX file, 0.1 MB. Copyright © 2019 Shoukat et al. 2019 Shoukat et al. This content is distributed under the terms of the Creative Commons Attribution 4.0 International license.

**FIG 2:**
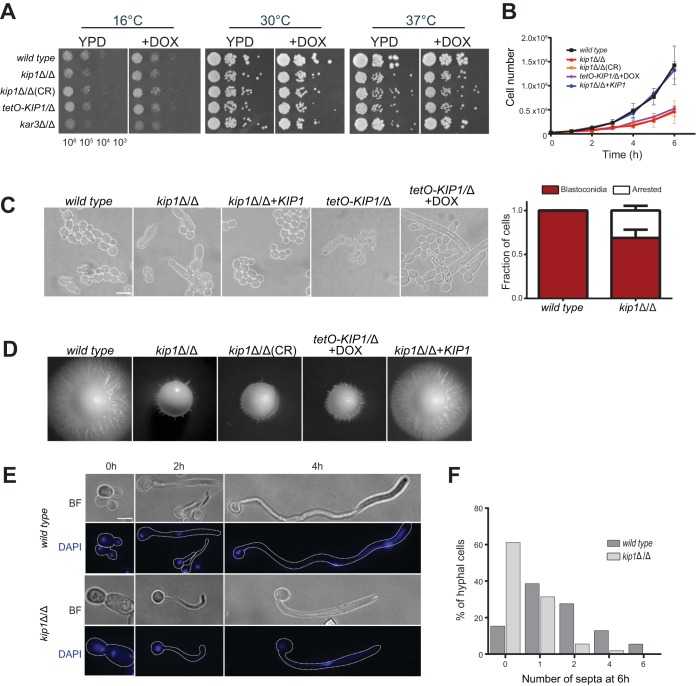
Loss of Kip1 affects growth and viability. (A) Spot assay of the various mutant *CaKIP1* strains to assess cell growth. *kip1*Δ/Δ, strain CF311; *kip1*Δ/Δ(CR) strain CF429; *tetO-KIP1*/Δ, strain CF436. Cells were serially diluted to the specified concentrations, and 5-μl droplets were plated on YPD with or without DOX (10 μg/ml). Plates were incubated for 2 days at the indicated temperatures. (B) Cell growth assay of the independent *CaKIP1* strains, including a *KIP1* add-back strain (CF354). Cells in SDC medium were diluted to 2.5 × 10^6^ cells per ml, incubated at 30°C, and counted every hour with a hemocytometer. Data points represent the average from three independent experiments ± standard error of the mean (SEM). (C) Cells were grown in SDC, and bright-field images were collected. The graph shows the proportion of normal-looking blastoconidia and arrested cells observed in these bright-field images. Data represent the average from three independent experiments ± SEM. *n* > 3,000 cells per strain. (D) Assessment of hyphal growth on various *CaKIP1*-null strains. The wild type and each *KIP1* mutant were plated onto Spider medium and incubated for 5 days at 30°C before imaging. (E) Bright-field (BF) and fluorescence images of wild-type and *kip1*Δ/Δ cell cultures. Cells were diluted 1:50 into fresh YPD medium supplemented with 10% FBS and incubated at 37°C to induce hyphae. Cells were removed from the cultures at the indicated time points and then fixed and stained with DAPI (4′,6-diamidino-2-phenylindole) before imaging. (F) Cells were induced to form hyphae under the same conditions in panel E and then fixed and stained with calcofluor white before imaging. The number of septa per hyphae was quantified and graphed.

In dilution spot assays, all *Ca*Kip1-depleted strains displayed modest sensitivity to higher temperature, but were otherwise viable (Fig. 2A). However, in liquid culture, cells lacking *Ca*Kip1 activity proliferated slower than wild-type cells and contained a mixture of blastoconidia and cells with long extensions resembling pseudohyphae (Fig. 2B and C). Upon further visual inspection and quantification of the *kip1*Δ/Δ strain by microscopy, we observed this hyperpolarized morphology in approximately 30% of the cells (Fig. 2C). These elongated cells indicate a delay in cell cycle progression or a cell cycle arrest and could mask a slower proliferation rate on solid growth medium by giving *kip1*Δ/Δ dilution spots a similar appearance to the wild type. We also found that loss of *Ca*Kip1 affected filamentous growth under hypha-inducing conditions. *Ca*Kip1-depleted colonies formed a smaller halo of invasive growth on Spider medium (Fig. 2D), and cells grown in serum produced shorter germ tubes and fewer septa (Fig. 2E and F). When we added a wild-type copy of *CaKIP1* back into the *kip1*Δ/Δ strain at the native locus, normal cell growth rate and cell morphology were restored (Fig. 2B to D), confirming that these defects were a direct consequence of *CaKIP1* loss.

**FIG 3:**
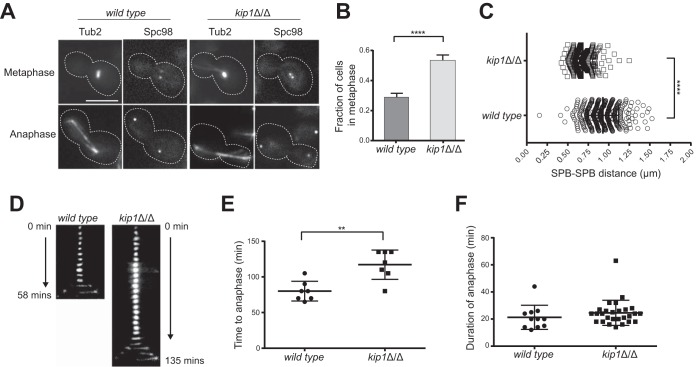
*kip1*Δ/Δ cells form bipolar spindles but exhibit defects in spindle dynamics. (A) Static images of wild-type (CF363) and *kip1*Δ/Δ (CF368) cells expressing Tub2-mCherry and Spc98-GFP in SDC-sucrose at 30°C. Scale bars, 5 μm. (B) Quantification of nuclear MT structures observed in Tub2-GFP-labeled wild-type (CF289) and *kip1*Δ/Δ (CF226) cells. The proportions of cells with metaphase spindles in *kip1*Δ/Δ cells were significantly different from that of wild-type cells (*P < *0.0001, Student’s test). Data represent mean values from three independent replicates of >1,000 cells for each genotype ± SEM. (C) The distance between SPBs in blastoconidia with a bipolar spindle was measured in logarithmically growing cells (wild type [CF363], *n* = 357; *kip1*Δ/Δ mutant [CF368], *n* = 367) ± SEM (*P < *0.0001, Student’s test). (D and E) Quantification of wild-type and *kip1*Δ/Δ cells using time-lapse microscopy (*n* = 7) (*P = *0.0019, Student’s test). (F) Quantification of wild-type and *kip1*Δ/Δ cells using time-lapse microscopy to analyze the duration of anaphase. Long (2 to 4 h) time-lapse series were captured with 150-ms exposures to measure the length of time from emergence of the bud until the end of anaphase for Tub2-GFP (*n* = 11) and Tub2-GFP *kip1*Δ/Δ (*n* = 28) cells (*P > *0.3, Student’s test).

Expecting that the slow-growth phenotype of *Ca*Kip1-depleted cells was caused by errors in mitotic spindle assembly, we imaged fields of unsynchronized wild-type and *kip1*Δ/Δ blastoconidia expressing Tub2-mCherry and Spc98-GFP (a component of the spindle pole body) and examined their spindle structures. We found that most of the spindles in budded *kip1*Δ/Δ cells (∼92%) formed a stereotypical bipolar spindle structure (Fig. 3A). However, nearly twice as many *kip1*Δ/Δ cells had metaphase spindles (53.5%) compared to the wild type (28.9%) (Fig. 3B), and the mean length of *kip1Δ*/*Δ* metaphase spindles was significantly shorter (*kip1Δ*/*Δ*, 0.68 ± 0.01 μm; wild type, 0.93 ± 0.01 μm) (Fig. 3C). When we tracked progression of the mitotic phases by time-lapse microscopy, we observed that *kip1Δ*/*Δ* cells took an average of 117.1 ± 7.8 min to initiate anaphase after a spindle had formed, whereas wild-type cells required only 80.0 ± 5.2 min on average (Fig. 3D and E). In contrast, when *kip1Δ*/*Δ* spindles did eventually elongate, there was no difference in the duration of anaphase compared to the wild type (Fig. 3F). These data show that C. albicans is not solely dependent on kinesin-5 activity for bipolar spindle assembly or late anaphase spindle elongation, but *Ca*Kip1 is important for timely separation of spindle poles after spindle assembly.

**FIG 4:**
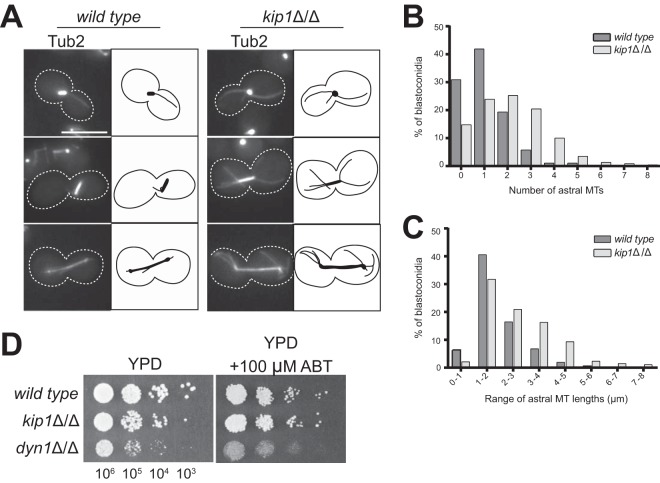
*kip1*Δ/Δ cells have longer and more numerous cytoplasmic MTs. (A) Representative images of wild-type and *kip1*Δ/Δ cells expressing Tub2-GFP are shown beside cartoon representations of each cell to illustrate the difference in astral MT numbers and lengths. (B) The number of astral MTs in wild type (CF289) and *kip1*Δ/Δ (CF226) expressing Tub2-GFP was counted in cells with visible spindles. (C) For cells in panel B that contained astral MTs, astral MT length was determined by measuring the distance between the metaphase spindle pole and the plus end. These lengths were organized into bins of the size ranges indicated. (D) Wild-type (CF027), *kip1*Δ/Δ (CF311), and *dyn1*Δ/Δ (CF358) cells were serially diluted to the indicated concentrations, and 5-μl droplets were plated on solid YPD medium and YPD plus 100 μM ABT and incubated for 2 days at 25°C.

Interestingly, *kip1Δ*/*Δ* spindles had longer and more numerous astral MTs than the wild type (Fig. 4A to C). In many eukaryotes, including C. albicans, the plus ends of astral MTs strike the cell cortex, where they are captured by the minus-end-directed MT motor protein dynein (54–56). When this happens, dynein can draw the MT, and the attached SPB, toward the cortical contact site to facilitate proper spindle positioning, elongation, and/or migration (57, 58). In S. cerevisiae, this activity of dynein assists Cin8 and Kip1 in anaphase spindle elongation, and simultaneous loss of dynein and Cin8 activity is lethal (22, 31). We found the *dyn1Δ*/*Δ* strain to be nonviable in the presence of the *Ca*Kip1-specific inhibitor aminobenzothiazole (ABT), suggesting that *Ca*Kip1 and dynein also have overlapping functions in C. albicans (Fig. 4D). Perhaps the longer and more numerous astral MTs in the *kip1Δ*/*Δ* strain are an adaptation to *Ca*Kip1 loss that provides more opportunities for MT capture and pulling events by dynein, which could promote both anaphase spindle elongation and SPB separation during spindle assembly.

### A subpopulation of *kip1*Δ/Δ cells have multiple spindles and show atypical cell cycle dynamics.

**FIG 5:**
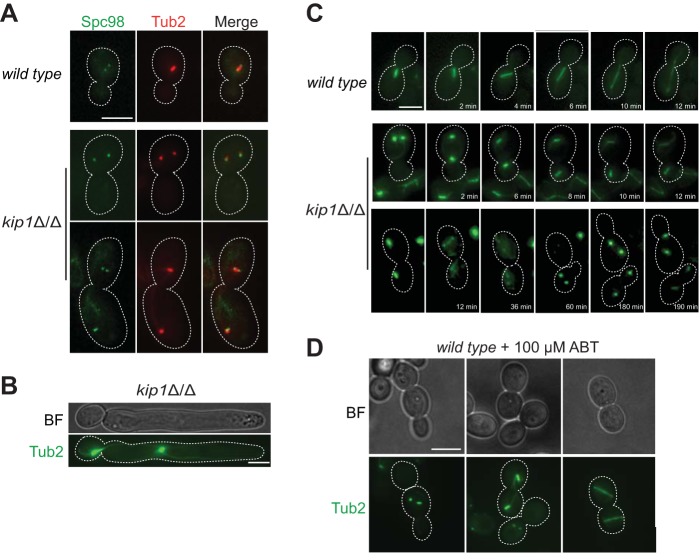
*kip1*Δ/Δ cells have abnormal number of spindles and SPBs. (A) Wild-type (CF363) and *kip1*Δ/Δ (CF368) cells expressing Tub2-mCherry and Spc98-GFP were grown in SDC-sucrose at 30°C. The top row shows a normal metaphase spindle in the wild type. The middle row shows two monopolar spindles in the mother compartment of the *kip1*Δ/Δ mutant. The bottom row shows a *kip1*Δ/Δ cell with one monopolar and one bipolar spindle. (B) Tub2-GFP-labeled *kip1*Δ/Δ (CF226) arrested cells display multiple spindles. (C) Time-lapse of wild-type (CF289) and *kip1*Δ/Δ (CF226) cells expressing Tub2-GFP. Two examples of *kip1*Δ/Δ spindle dynamics are shown (rows 2 and 3). (D) Inhibition of *Ca*Kip1 by ABT phenocopies *kip1*Δ/Δ cells. Tub2-GFP wild-type cells (CF289) were incubated with 100 μM ABT for 2 h and imaged. Scale bar, 5 μm.

Similar to previous findings by Chua et al. (42), we observed that a significant proportion (∼12%) of *kip1*Δ/Δ blastoconidia and all *kip1*Δ/Δ cells with a hyperelongated morphology, contained multiple spindles (Fig. 5A and B). In some cases, monopolar and bipolar spindles were simultaneously visible within the same budding cell (Fig. 5A, row 3). To determine how these extra spindles formed, we collected time-lapse images of *kip1*Δ/Δ blastoconidia expressing Tub2-GFP. All of the multispindle blastoconidia that we tracked (*n* = 20) formed two short “bars” of tubulin fluorescence in the mother cell as the new bud began to emerge. Once a new bud formed, we observed two different multispindle configurations. In 65% of the cells we imaged, one of the tubulin structures traversed the bud neck, while the other remained in the mother compartment (Fig. 5C, row 2, *t* = 6 min). Each short fluorescent bar then elongated simultaneously, suggesting that they had formed distinct bipolar spindles. However, both spindles broke apart or disintegrated once the cell divided (Fig. 5C, row 3, t = 36 min; see Movie S1 in the supplemental material). This phenotype suggests that *Ca*Kip1 activity is important for anaphase spindle stability. In a smaller cohort of cells (45%), one or both of the spindles elongated across the bud neck and appeared to complete anaphase (not shown). Both of these multispindle phenotypes were recapitulated in wild-type cells treated with ABT (Fig. 5D).

10.1128/mSphere.00610-19.2 MOVIE S1 Loss of Ca*KIP1* results in multiple spindles. Time-lapse microscopy of *kip1*Δ/Δ (CF226) cells expressing Tub2-GFP. Frames were captured at 2-min intervals for a duration of 3 h and 30 min at 30°C. The GFP channel is a maximum projection of 5 z-planes, 0.8 μm apart using 100-ms exposures at 25% LED output. Running time is in minutes at 3 frames per second. Download Movie S1, AVI file, 3.0 MB. Copyright © 2019 Shoukat et al. 2019 Shoukat et al. This content is distributed under the terms of the Creative Commons Attribution 4.0 International license.

**FIG 6:**
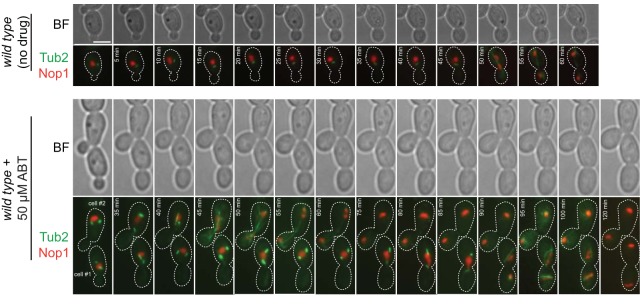
A subpopulation of *kip1*Δ/Δ cells display abnormal nuclear division. (Rows 1 and 2) Time-lapse microscopy of wild-type cells expressing Nop1-mScarlet and Tub2-GFP (CF417) (the “no-drug” condition). (Rows 3 and 4) Wild-type cells expressing Nop1-mScarlet and Tub2-GFP were incubated with 50 μM ABT for 3 h and imaged every 5 min. Exposure times were 150 ms. Scale bar, 5 μm.

We were intrigued by this spindle defect because a subpopulation of wild-type C. albicans cells exposed to the antifungal agent ﬂuconazole (FLC) display abnormal numbers of spindles as well. In the presence of FLC, DNA replication and nuclear division proceed ahead of bud emergence and completion of cytokinesis, respectively. Harrison et al. (59) showed that when this happens, some nuclei re-fuse or fail to separate due to mitotic collapse, forming tetraploid progeny with extra spindle components. Therefore, we next used time-lapse microscopy to track nucleolar segregation (using Nop1-mScarlet) in ABT-treated wild-type cells that formed multiple spindles. While the “no-drug” condition showed stereotypical nuclear and spindle dynamics that were well coordinated with bud emergence and growth (Fig. 6, rows 1 and 2; see Movie S2 in the supplemental material), ABT-treated cells contained one large patch of Nop1-mScarlet fluorescence and two separate bars of Tub2-GFP when the bud emerged (Fig. 6, row 4; see Movie S3 in the supplemental material). This indicates that a bipolar spindle had already formed and broken apart before bud evagination. As bud growth continued, the Nop1 patch divided and migrated with each spindle. When these spindles were segregated to the mother and daughter cell (cell 1, 65% of the cells imaged), two smaller nuclear fragments were visible in each compartment after spindle elongation (four Nop1-mScarlet patches in total, cell 1, *t* = 95 min). However, within 20 min, each pair of Nop1 patches coalesced as a result of mitotic collapse. When the spindles elongated across the neck (cell 2, 45% of the cells imaged), each spindle divided a Nop1-mScarlet patch into two different pieces, again resulting in four separate nuclear fragments (cell 2, *t* = 45 min). Here, the two Nop1 foci in each compartment were produced from different anaphase spindles. As the time-lapse continued, these Nop1 foci appeared to merge into one (Fig. 6, cell 2, t = 75 min). These results suggest that the extra spindles seen in *kip1*Δ/Δ cells and ABT-treated wild-type cells could have formed in nuclei that experienced mitotic collapse or after merging of nuclear fragments from two distinct spindles that completed anaphase.

10.1128/mSphere.00610-19.3 MOVIE S2 Wild-type cells display normal nuclear division. Time-lapse microscopy of wild-type cells expressing Nop1-mScarlet and Tub2-GFP (CF417). Frames were captured at 5-min intervals for a duration of 1 h and 10 min at 30°C. GFP and TRITC channels are maximum projections of 5 z-planes, 0.8 μm apart using 100-ms exposures at 95% LED output. The running time is in minutes at 3 frames per second. Download Movie S2, AVI file, 0.1 MB. Copyright © 2019 Shoukat et al. 2019 Shoukat et al. This content is distributed under the terms of the Creative Commons Attribution 4.0 International license.

10.1128/mSphere.00610-19.4 MOVIE S3 *kip1*Δ/Δ cells display abnormal nuclear division. Wild-type cells expressing Nop1-mScarlet and Tub2-GFP were incubated with 50 μM ABT for 3 h and imaged every 5 min. Exposure times were 150 ms. Scale bars, 5 μm. Images are captured and processed as in Movie S2, but with a 3-h duration. Running time is in minutes at 3 frames per second. Download Movie S3, AVI file, 0.3 MB. Copyright © 2019 Shoukat et al. 2019 Shoukat et al. This content is distributed under the terms of the Creative Commons Attribution 4.0 International license.

### Simultaneous loss of *Ca*Kip1 and *Ca*Kar3/Cik1 function is lethal.

**FIG 7:**
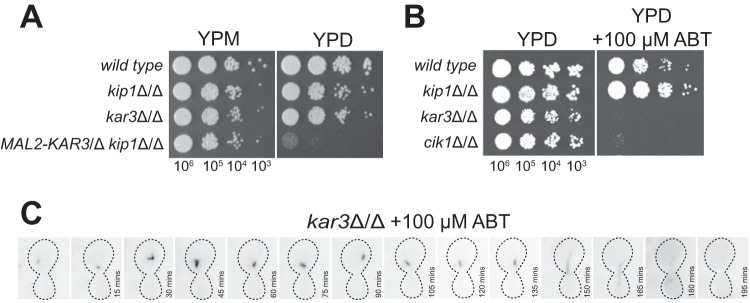
Loss of kinesin-5 and kinesin-14 function is lethal. (A) Wild-type (CF027), *kip1*Δ/Δ (CF311), *kar3*Δ/Δ (CF024), and *MAL2*-*KAR3*/Δ *kip1*Δ/Δ (CF396) cells were plated on YPM or YPD. Cells were serially diluted to the indicated concentrations, and 5-μl droplets were plated and incubated for 2 days at 25°C. (B) The strains of the genotypes indicated in panel A, in addition to *cik1*Δ/Δ (CF016), were plated on YPD or YPD plus 100 μM ABT and were plated as in panel A. (C) Time-lapse microscopy of ABT-treated *kar3*Δ/Δ cells (CF172). Cells were imaged every 15 min with a 150-ms exposure time to avoid photobleaching. Between 165 and 180 min, the short anaphase spindle breaks down and disappears.

In many of the eukaryotic systems, inactivation of kinesin-14 rescues the lethal spindle defects arising from inhibition or loss of kinesin-5 activity and spindles recover the ability to complete a relatively normal mitotic cycle (12–16, 37, 38, 60–65). This has been rationalized as a restoration of force balance in the spindle, where compensatory spindle forces are provided by MT polymerization and cross-linking proteins (17, 34, 66). In spite of repeated attempts, we were unable to obtain a *kip1*Δ/Δ *kar3*Δ/Δ strain by traditional methods (data not shown), suggesting they are synthetically lethal. To confirm this, we constructed a *KIP1* knockout strain containing only one functional copy of *KAR3* that is under the control of the maltose-inducible Mal2 promoter. Indeed, when we deactivated the Mal2 promoter by culturing this strain on glucose (YPD [yeast extract-peptone-dextrose] medium), cell growth was arrested, demonstrating that simultaneous loss of *Ca*Kar3 and *Ca*Kip1 is lethal (Fig. 7A). We also observed that *kar3Δ*/*Δ* and *cik1*Δ/Δ cells were not viable in the presence of ABT (Fig. 7B). To visualize events leading up cell death by loss of *Ca*Kip1 and *Ca*Kar3 function, we imaged Tub2-GFP fluorescence in *kar3*Δ/Δ cells treated with 100 μM ABT every 15 min by time-lapse microscopy to avoid photobleaching. After 165 min of imaging, we observed a short anaphase spindle that did not elongate further. Within 3 h, spindle structures disappeared and cells showed no tubulin fluorescence (Fig. 7C). These results demonstrate that kinesin-5- and kinesin-14 have more functional overlap in C. albicans than in other organisms.

## DISCUSSION

In nearly every type of eukaryotic system studied, kinesin-5 activity is needed to push newly duplicated centrosomes or SPBs apart to establish spindle bipolarity (1–4, 8, 23). Kinesin-5s also cross-link and bundle parallel and antiparallel spindle MTs and are the major providers for outward forces during anaphase spindle elongation (1–4, 7, 8, 23, 67). Without them, most cells exhibit mono-astral spindles and are nonviable (2, 9–11). In contrast, our genetic data demonstrate that the sole kinesin-5 gene in C. albicans is not essential in diploid cells. Perhaps the recent finding that *CaKIP1* is an essential gene in a haploid isolate of C. albicans (51) is an indication that *CaKIP1* mutants exhibit a form of ploidy-specific lethality, which is shared by other yeast genes involved in mitotic spindle stability (68). An alternative explanation for this discrepancy is that there are differences in protein expression between the haploid and diploid proteomes (69). Our results further show that kinesin-5 is dispensable for spindle assembly and anaphase spindle elongation in C. albicans. The only other organisms reported to complete mitosis without kinesin-5 activity are Caenorhabditis elegans and Dictyostelium discoideum (70–72). Although their mechanisms for kinesin-5-independent bipolar spindle assembly and elongation are not yet known, it has been suggested that cytoplasmic dynein-mediated astral MT pulling forces are involved. We propose that dynein may also fulfill these roles in C. albicans in the absence of kinesin-5 activity for several reasons. In earlier studies, C. albicans cells lacking the heavy chain of cytoplasmic dynein or the p150^Glued^ subunit of dynactin exhibited spindle position, orientation, and elongation defects, and dramatically slowed nuclear dynamics (73, 74). In the filament-forming fungus Ustilago maydis, it was shown that stationary dynein motors capture and pull on the plus ends of astral MTs that emanate from SPBs, drawing the attached SPB toward the cortical contact site (75). In our studies, astral MTs were much longer and more numerous in *kip1Δ*/*Δ* cells, which could increase the frequency of these MT capture and dynein-mediated pulling events for SPB separation and spindle elongation. We also found *dyn1Δ*/*Δ* mutants to be nonviable in the presence of the inhibitor ABT, suggesting that *Ca*Kip1 and dynein have overlapping functions. Our future studies aim to better understand this putative intersection of dynein and *Ca*Kip1 functions. We will also investigate the alternative possibility that *Ca*Kip1 has a direct role in limiting the number and length of astral MTs, based on recent evidence that kinesin-5s can act as length-dependent MT depolymerases at kinetochores (46, 76, 77).

Although *kip1*Δ/Δ cells readily assembled metaphase spindles, these spindles were shorter and delayed in transitioning to anaphase relative to wild-type cells. This suggests a role for *Ca*Kip1 in maintenance of the bipolar spindle prior to anaphase, which is an important kinesin-5 function in other fungi and in *Xenopus* and *Drosophila* (3, 4, 45, 78). The redistribution of *Ca*Kip1-GFP fluorescence along the spindle in early anaphase supports such a role. The metaphase-to-anaphase delay in *kip1*Δ/Δ cells may also explain their lower growth rate in liquid cultures. Our observation that *kip1*Δ/Δ spindles sometimes broke apart and then reassembled two new bipolar spindles also supports this function and implies that *Ca*Kip1 is acting as a MT cross-linker within the spindle. An alternative explanation for these short, unstable spindles is that *Ca*Kip1 regulates kMT dynamics, which is important for congression of bioriented sister chromosomes in metaphase. Indeed, S. cerevisiae*’s* Cin8 is important for kinetochore clustering/positioning near the SPBs by cross-linking kMTs and promoting the disassembly of long kMTs (28, 46). In S. pombe, Cut7 is recruited to the kinetochores by a spindle assembly checkpoint (SAC) protein, Mad1, to promote chromosome gliding toward the spindle equator (29). Recent EM reconstructions of C. albicans *KIP1*/*kip1Δ* spindles show disorganized kMTs (76), suggesting that chromosomes are not properly congressed at the spindle equator during metaphase. Without proper chromosome congression, mitotic errors are more likely to occur. Perhaps the short bipolar spindles we observed in *kip1*Δ/Δ cells are indicative of attempts to correct erroneous kMT attachments (76), and spindle disassembly occurs when they are not corrected. If these cells initiate DNA replication and attempt mitosis again, this could explain the extra spindles and nucleoli observed in a subpopulation of C. albicans *kip1*Δ/Δ cells. Further work will be needed to uncover whether or not these defects in nuclear dynamics lead to an increase in the prevalence of aneuploid cells.

Surprisingly, *Ca*Kip1 loss did not extend the duration of anaphase relative to wild type, even though *Ca*Kip1-GFP accumulated at the midzone of late anaphase spindles; a site where it could exert outward MT sliding forces for spindle elongation. This is unique from other fungi and *Drosophila* embryos, which rely on kinesin-5 to cross-link overlapping antiparallel MTs in the spindle midzone and drive anaphase B spindle elongation via plus-end-directed motility (6, 17, 22, 30–34, 63). While we suspect that dynein provides pulling forces on the spindle to assist in anaphase spindle elongation in the absence of *Ca*Kip1, it is also possible that other kinesins or MT cross-linking components within the spindle are involved. In fission yeast, kinesin-6 provides additional MT-sliding forces to kinesin-5 at the spindle midzone for anaphase spindle elongation and dynein is not involved (17, 66). Although C. albicans has no kinesin-6 homolog in its genome, it encodes five other kinesin-like proteins in addition to *Ca*Kip1. Therefore, we have begun to generate strains lacking different combinations of these proteins in order to identify new collaborative roles of kinesins in mitosis.

By simultaneously disrupting kinesin-5 and kinesin-14 activities, we found that C. albicans displays a puzzling exception to the widely regarded spindle force-balance model (15, 37, 62, 79). Rather than providing antagonistic spindle forces, *Ca*Kip1 and *Ca*Kar3Cik1 may cooperate to focus and stabilize parallel and antiparallel interactions in certain areas of the spindle. In this regard, loss of both kinesins may reduce the number of MT cross-linking factors to an intolerable level that cannot support cell viability. Our previous finding that *Ca*Kar3Cik1-depleted cells often arrest with two monopolar half-spindles that become pulled apart before assembling a bipolar spindle, supports this idea (41). Combined with *Ca*Kip1 loss, MTs may not be well tethered at SPBs or fail to focus kinetochores, resulting in disorganized spindle structures that quickly break down.

C. albicans is a close relative of the model yeasts S. pombe and S. cerevisiae but is also an opportunistic fungal pathogen. An assortment of fitness attributes promote its pathogenicity (80), most of which arise by rapid genetic diversification within a population in response to stressful growth conditions as a means of adaptation (81, 82). Research has shown that aneuploidy accounts for much of this diversity (83–85), and recent findings suggest that aneuploidies could be induced or enabled by altered activity of mitotic kinesin motors under stress (39, 49, 86–88). As specific aneuploidies can confer resistance to antifungal drugs through altered gene copy numbers, it could be advantageous for C. albicans cells to regulate mitotic kinesins as a way to control aneuploidy occurrence. We are currently conducting studies to delineate the putative contributions of C. albicans kinesins to mitotic defects of cells under stress and to identify stress-specific regulatory factors that change kinesin activity to promote aneuploidy.

## MATERIALS AND METHODS

### Genetic manipulations.

A list of C. albicans strains used in this study is presented in Table 1. The oligonucleotides used in strain construction are listed in Table 2. Gene disruption of the C. albicans *KIP1* open reading frame (*Candida* Genome Database, *orf19.8331*; NCBI Gene ID, 3645256) was conducted by PCR-based gene targeting and CRISPR-Cas9 methods (89). PCR amplification was used to generate disruption cassettes where a selectable marker was flanked by approximately 50 bp of C. albicans genomic sequence immediately 5′ and 3′ of the *KIP1* coding region. Disruption of *KIP1* in a wild-type strain (CF027) was conducted sequentially. First a *kip1*::*LEU2^+^* cassette was amplified from pSN40 (90) using primers P118 and P119 and transformed into strain CF027. Correct *kip1*::*LEU2^+^* cassette integration was confirmed using primer pairs P120/P13 and P121/P14 for the upstream and downstream junctions, respectively. To disrupt the second *KIP1* allele, a *kip1*::*HIS1^+^* cassette was amplified from pSN52 (90) using primer pair P118/P119 and transformed to create strain CF311. Integration of the disruption cassette at the correct location was confirmed by PCR amplification across the junctions of integration using primers P120/P11 and P121/P12 for the upstream and downstream regions, respectively. CRISPR-Cas9-mediated *kip1* deletion was conducted as previously described (89) using the custom guide RNA (gRNA) primer P247 and double-stranded donor DNA formed using primers P248/P249 to create the strain CF429 (89). To regulate the expression of *KIP1*, the tetracycline-repressible transactivator, the *tetO* promoter, and the NAT flipper cassette were PCR amplified from pLC605 (kindly provided by Leah Cowen) using primers P240/P241. The PCR-amplified product was transformed into the heterozygous *KIP1* strain to create strain CF436. Correct integration at the *KIP1* locus was verified using primer pairs P120 and P242.

**TABLE 1:** Names, genotypes, mating types, and sources of the strains used in this study^*a*^

Strain	Genotype		Mating type	Source or reference
	Brief description	Full description		
CF027	Wild type	*his1^−^*/*his1^−^ leu2^−^*/*leu2^−^ arg4^−^*/*arg4^−^*	α/α	RBY1133 (98)
CF016	*cik1*Δ/Δ	*cik1*Δ::*LEU2^+^*/*cik1*Δ::*HIS1^+^*	α/α	41
CF024	*kar3*Δ/Δ	*kar3*Δ::*LEU2^+^*/*kar3*Δ::*HIS1^+^*	α/α	RSY11 (98)
CF311	*kip1*Δ/Δ	*kip1*Δ::*LEU2^+^*/*kip1*Δ::*HIS1^+^*	α/α	CF236 (this study)
CF429	*kip1*Δ/Δ (CR)	*kip1*Δ::gRNA (see Table 2)	α/α	AHY940 (89)
CF436	*tetO-KIP1/*Δ	*tetO-KIP1*/*kip1*Δ::*LEU2^+^*	α/α	CF236
CF354	*kip1*Δ/Δ/ *KIP1^+^*	*kip1*Δ::*HIS1^+^*/*kip1*Δ::*LEU2^+^*::*KIP1-ARG4^+^*	α/α	CF311 (this study)
CF338	pGAL1-Tub2-mCherry KIP1-GFP	*NEUT5L*::[pGAL1-Tub2-mCherry*-SAT1*^R^]/*NEUT5L^+^* *KIP1-*GFP*-ARG4^+^*	α/α	CF306 (this study)
CF443	pGAL1-Tub2-mNeon KIP1-mScarlet	*NEUT5L*::[pGAL1-Tub2-mNeon*-ARG4^+^*]/*NEUT5L^+^* *KIP1-*mScarlet*-SAT1*^R^	α/α	CF421 (this study)
CF340	pGAL1-Tub2-mCherry *KIP1-*GFP *cik1*Δ/Δ	*NEUT5L*::[pGAL1-Tub2-mCherry*-SAT1*^R^]/*NEUT5L^+^* *KIP1-*GFP*-ARG4^+^ cik1*Δ::*LEU2^+^*/*cik1*Δ::*HIS1^+^*	α/α	CF308 (this study)
CF289	pGAL1-Tub2-GFP	*NEUT5L*::[pGAL1-Tub2-GFP*-SAT1*^R^]/*NEUT5L^+^*	α/α	CF027
CF226	pGAL1-Tub2-GFP *kip1*Δ/Δ	*NEUT5L*::[pGAL1-Tub2-GFP*-SAT1*^R^]/*NEUT5L^+^* *kip1*Δ::*ARG4^+^*/*kip1*Δ::*LEU2^+^*	α/α	CF311 (this study)
CF363	pGAL1-Tub2-mCherry *SPC98*-GFP	*NEUT5L*::[pGAL1-Tub2-mCherry*-ARG4*^R^]/*NEUT5L^+^* *SPC98-*GFP*-SAT1*^R^	α/α	CF156 (this study)
CF368	pGAL1-Tub2-mCherry *SPC98*-GFP *kip1*Δ/Δ	*NEUT5L*::[pGAL1-Tub2-mCherry-*ARG4^+^*]/*NEUT5L^+^* *SPC98-*GFP*-SAT1*^R^ *kip1*Δ::*HIS1^+^*/*kip1*Δ::*LEU2^+^*	α/α	CF286 (this study)
CF417	pGAL1-Tub2-GFP *NOP1-*mScarlet	*NEUT5L*::[pGAL1-Tub2-GFP*-ARG4^+^*]/*NEUT5L^+^* *NOP1-*mScarlet*-SAT1*^R^	α/α	CF405 (this study)
CF396	*MAL2-KAR*/Δ *kip1*Δ/Δ	*kar3*Δ::*HIS1^+^*/*KAR3-MAL2-ARG4^+^ kip1*Δ::*LEU2^+^*/*kip1*Δ::*SAT1*^R^	α/α	CF388 (this study)
CF358	*dyn1*Δ/Δ	*dyn1*Δ::*URA3^+^/dyn1*Δ::*HIS1^+^ Hhf1-*GFP*-Arg*	α/α	73
CF172	pGAL1-Tub2-GFP *kar3*Δ/Δ	*NEUT5L*::[pGAL1-Tub2-GFP*-SAT1*^R^]/*NEUT5L^+^* *kar3*Δ::*HIS14^+^/kar3*Δ::*LEU2^+^*	α/α	41

a Strains are in the white phase unless otherwise noted. All strains are derived from SN152 (90). The full genotype at auxotrophic markers is *his1*::*hisG*/*his1*::*hisG leu2*::*hisG*/*leu2*::*hisG arg4*::*hisG*/*arg4*::*hisG*/*ura3*::*imm434*::*URA3*/*ura3*::*imm434 iro1*::*IRO1*/*iro1*::*imm434*.

**TABLE 2:** Oligonucleotide primers used in strain construction

Primer	Description	Sequence (5′ to 3′)^*a*^
P118	Long homologous tail knockout primer *KIP1*::*HIS1/LEU2/ARG4* 5′	GTTGTTGTTGTTTTCATTCTTCATCTTGTGATTTCAGTTAAATTAATACTCATA GCAGCATTATCATCA***ACCAGTGTGATGGATATCTGC***
P119	Long homologous tail knockout primer *KIP1::HIS1/LEU2/ARG4* 3′	AAATAAACCTCACAATTAATTAAACATGTACTGAACAAATGGAGTAAAACA AATATTGGTCTAATTATA***AGCTCGGATCCACTAGTAACG***
P120	−500 bp *KIP1* check 5′	CGCACAAGACCTGGCACAAGAGAA
P121	+500 bp *KIP1* check 3′	ATGGGCCAATGGGATCACATGG
P11	*HIS1* check right 3′	AACACAACTGCACAATCTGGC
P12	*HIS1* check left 5′	ATTAGATACGTTGGTGGTTCAGTT
P13	*LEU2* check left 3′	AGAATTCCCAACTTTGTCTGTTC
P14	*LEU2* check right 5′	AAACTTTGAACCCGGCTGCG
P247	*KIP1* gRNA for fragment B stitching	CGTAAACTATTTTTAATTTGCGAAGTAATACTGCTTGTGGGTTTTAGAGCTAG AAATAGC
P248	*KIP1* donor DNA with mini-AT 5′	ATTCTTCATCTTGTGATTTCAGTTAAATTAATACTCATAGCAGCggGACCAATA TTTGTTTTACTCCATTTGTTCAGTACATGTTTAATTAATTGT
P249	*KIP1* donor DNA with mini-AT 3′	ACAATTAATTAAACATGTACTGAACAAATGGAGTAAAACAAATATTGGTCccG CTGCTATGAGTATTAATTTAACTGAAATCACAAGATGAAGAAT
P240	*KIP1 tetO*-*SAT*^R^ flipper 5′	ATTCATTCATTCAATCAGAGTAGTTTTAATATCTTCTTATAGTGGCCTGCATAT AGTTCAATCACGAC***GGAAACAGCTATGACCATG***
P241	*KIP1 tetO-SAT*^R^ flipper 3′	GAGATTTAGCAGCAATCTCTTGAGAGTTCCTTCCTCGACATCTAACAACAAC TTGGATATTTGACCGCGG***CGACTATTTATATTTGTATG***
P242	*tetO* check 3′	AGTTATTGAATCTATTACTCAATCG
P170	*KIP1* ORF confirmation primer 3′	CTTCATTCACTATATTTCCAACTTGTGATTG
P128	*KIP1* into pCIp10 (MluI) primer 5′	GGACCGACGCGTCACAGAGAGAGAGAGAGAGAGAAAGAGAATGAG
P129	*KIP1* into pCIp10 (KpnI) primer 3′	GGACCGGGTACCCATCATCAACATAATCAACCACATCACCCACA
P199	Long homologous tail knockout primer *KAR3*::*HIS1*/*LEU2*/*ARG4* 5′	TCAAAAAGTTGCCAGACAGGTTTTTTACAATTTTGAAACTACAATCCAATAG TCAATCGTGCACAAGTA***ACCAGTGTGATGGATATCTGC***
P200	Long homologous tail knockout primer *KAR3::HIS1^+^*/*LEU2^+^*/*ARG4^+^* 3′	TATATCTGAGCCAATATTTAAATAGATTCTTGTATATAAGTCATGTATGTAAAC TATTAACGTAGTAAT***AGCTCGGATCCACTAGTAACG***
P201	−1,000 bp *KAR3* check 5′	GTCCCAACTTCTCCTTATTGACTTCTT
P202	+1,000 bp *KAR3* check 3′	GTTGCCTAAAATTCCTAAGGACCT
P212	*ARG4-MAL2-KAR3* long homologous primer 5′	AAAGAAAAACTTGCCCATCTCATCGAGAGTCTAATTTCTTACGCGGGAACTAG AAAAAAAAAACTGAA***GAAGCTTCGTACGCTGCAGGTC***
P213	*ARG4-MAL2-KAR3* long homologous primer 3′	CCACCTAAAAGATTTGATGGTTGTGACACATTTAGAAATTTATGTTTAGTATTT TCGTCACTCAT***TGTAGTTGATTATTAGTTAAACCAC***
P16	*ARG4* check left 3′	TTCCATTTAGAGAAACTCATCATATTT
P17	*SAT1*^R^ check left 3′	CATACCACCGTCCATTTTGAATG
P18	*SAT1*^R^ check right 5′	TGATGAAGACTCTGCTTGCTATG
P137	*KIP1-*GFP*-ARG4* or *SAT1*^R^ long-tailed primer (C terminal) 5′	TTCTACCACGACCAATAATAATAAAAAGAGAAAAATATTACAAACAATGGAC AATTTATTA***GGTGGTGGTTCTAAAGGTGAAGAATTATT***
P187	*KIP1-*GFP*-ARG4* or *SAT1*^R^ long-tailed primer (C terminal) 3′	CATATATTATATATTAATATTATTAAGAGTTTTTGGAAATATGGAACTATAAT GAGG***AGGACCACCTTTGATTGTAAATAGTAATAATTA***
P69	GFP sequencing/left junction check 3′	GATCTGGGTATCTAGCAAAAC
P169	*KIP1* ORF confirmation primer 5′	GCACAAGTCAATCTACTGGAAACAT
P284	*KIP1-*mScarlet localization long-tail 5′	TGTTGTTGTTTTCATTCTTCATCTTGTGATTTCAGTTAAATTAATACTCATAGC AGCATTATCATCA***GACTCACTATAGGGCGAATTGGG***
P285	*KIP1-*mScarlet localization long-tail 3′	AAATAAACCTCACAATTAATTAAACATGTACTGAACAAATGGAGTAAAACA AATATTGGTCTAATTATA***CAAAAGCTGGAGCTCCACCGC***
P254	mScarlet check 5′	GTAGATATTTGGCTGATTTCAAAAC
P108	*SPC98*-*GFP*-*SAT1*^R^ long-tailed primer for pGFP-SAT1 5′	TTTGAAAAATGATTTGAATAGAGATTATAATTTAAAGGATCTTAGTAAGTTGTT GGTGGTGGT***TCTAAAGGTGAAGAATTATTCACTGG***
P109	*SPC98*-GFP*-SAT1*^R^ long-tailed primer for pGFP-SAT1 3′	TGAGCTTTACAGAGATCTTGTCGGTAATCATAGATTTCCCCACTTGTTCTGTAA TCGACGAAATTG***AGGACCACCTTTGATTGTAAATAG***
P110	*SPC98-*GFP integration check 3′	GCAGCGTCCACCCTTTGTAAAAGTG
P107	pGAL1-Tub2-GFP/mCherry/mNeon downstream check 3′	TATTATCTATATTGTCAAGCCAAGACAAGCCCATT
P243	*NOP1-*mScarlet long-tail 5′	ACCTTATGAAAGAGACCATTGTATTGTTGTTGGTAGATACATGAGAAGCGGA ATAAAGAAAGGTGGTAGTGGTATGGTTTCTAAAG
P244	*NOP1-*mScarlet long-tail 3′	AAGGTCAAAGTGCCATCAAAGGTGTGTTATTGGGTTCATTATCAAATTATTTG GTGACAA***GGCGGCCGCTCTAGAACTAGTGGATC***
P246	*NOP1* check 3′	CGATTGAACATGTTAAACAAAGC

a The portion of primer homologous to plasmid template is in boldface and italic. The restriction enzyme cut site is underlined. Lowercase letters represent the mini-ADD-TAG sequence (mAT [gg]) for subsequent CRISPR-mediated gene editing (89).

To demonstrate that mutant phenotypes are solely a result of loss of *KIP1*, add-back strains were created to reintroduce a wild-type copy of each gene. The *KIP1* gene (±1,000 bp upstream/downstream) was cloned into pCIp10-based integration plasmids bearing the *ARG4^+^* selectable marker using primers P128/P129 (41, 91). The integration plasmid was digested at a unique restriction site (PmlI) to add back to the endogenous *KIP1* region into CF311 to create CF354. Confirmation of integration of the pCIp10*-ARG4^+^* vector was done using P121/P170.

A strain lacking both *Ca*Kip1 and *Ca*Kar3Cik1 function was created by deleting both copies of *KIP1* and one copy of *KAR3* and by placing the remaining functional *KAR3* copy under the control of a maltose promoter as follows: one copy of the *KAR3* ORF was disrupted using *kar3*::*HIS1^+^* knockout cassette amplified using primers P199/P200, transformed into CF027, and confirmed using primers P201/P11 and P202/P12 for the upstream and downstream junctions, respectively. The *ARG*::*MAL*::*KAR3* cassette was amplified using primer pair P212/P213 from pFA-ARG4-MALp (92) and transformed in *kar3*Δ::*HIS1^+^* to create CF411 (not shown). Integration of the cassette was confirmed using primers P201 and P16. *KIP1* was disrupted using LEU2 (described above) and the SAT1 nourseothricin resistance marker to create the strain CF396. Correct *kip1*::*SAT1* integration was confirmed with primer pairs P128/P17 and P129/P18.

Fluorescent tagging of *KIP1^+^* in wild-type cells was accomplished using the method described by Gerami-Nejad et al. (93) and using long-tailed primers P137 and P187 and the plasmid pGFP-SAT1 as a template to create an integration cassette bearing approximately 50 bp of *KIP1^+^* ORF immediately before the stop codon and of sequence 3′ to the ORF. This cassette was transformed into the wild type (CF027) to create the *KIP1-*GFP*-SAT1*^R^ strain. Correct integration was confirmed by PCR using the primer pair P69 and P169. The same integration cassette was also transformed in *cik1*Δ/Δ to create strain CF308 (not shown). *KIP1-*mScarlet was amplified using pScarlet plasmid pRB897 (kindly provided by Richard Bennett) using primers P284 and P285 and transformed into CF421 (pGAL1-Tub2-mNeon) to create CF443. Integration was confirmed using primers P254 and P121.

Strains expressing fluorescently labeled β-tubulin were constructed using the plasmids pGAL1-Tub2-GFP-SatR::NEUT5L, pGAL1-Tub2-mCherry-Arg4::NEUT5L, or pGAL1-Tub2-mNeon-Arg4::NEUT5L using the previously described method (41) and adapted to further include sequence of the neutral NEUT5L locus, which was linearized using the restriction enzyme KpnI. pGAL1-Tub2-GFP was transformed into CF027 and CF311 to create CF289 and CF226, respectively. pGAL1-Tub2-mCherry was transformed into the wild-type, *kip1*Δ/Δ, and *cik1*Δ/Δ strains to create CF363, CF368, and CF340, respectively. pGAL1-Tub2-mCherry was transformed into *KIP1*-*GFP* to create strain CF338. Correct integration for pGAL1 vectors was confirmed by PCR using primers P16 (or P17, depending on SAT/ARG markers) and P107. Induction of the GAL1 promoter, leading to expression of fluorescently tagged tubulin, was done by growing the cells in SDC-sucrose medium supplemented with 1% galactose. To visualize the nucleus, the nucleolar protein Nop1 was fluorescently labeled using pScarlet. The integration cassette was PCR amplified using primers P243 and P244 and transformed into CF289 to create CF417. Correct integration was confirmed by PCR using primers P254 and P246. To visualize spindle pole body structures, strains expressing *SPC98*-*GFP* were constructed as previously described (41).

### C. albicans transformation.

Disruption cassettes, fluorescent tags, and complementation plasmids were transformed into C. albicans using the lithium acetate-polyethylene glycol (PEG) heat shock method as previously described with minor modifications (94). Incubation of cells with transforming DNA in lithium acetate-PEG solution was carried out for 2 h at 30°C with rotation. Heat shock was conducted at 43°C for 30 min. Transformations involving selection using the *SAT1* gene were accompanied by a 4-h incubation in YPD (1% yeast extract, 2% peptone and 2% glucose) at 30°C to allow expression of the ClonNAT resistance gene before plating on selection medium.

### C. albicans cell culture and growth assays.

Strains were maintained on YPD plates. YPD was supplemented with 200 μg/ml nourseothricin (clonNat; Werner BioAgents) for selection of positive *SAT1* gene integration. Selection for auxotrophic markers was conducted using synthetic dropout (SD) medium containing 0.66% yeast nitrogen base, 0.2% yeast dropout mix lacking uracil, arginine, leucine, and histidine, 2% glucose, and 200 mg/liter uridine and supplemented with 200 mg/liter histidine, leucine, and/or arginine where required. Experimental cultures were grown to mid-logarithmic phase in completely supplemented dropout medium (SDC) unless otherwise indicated. In order to assess the generation time, logarithmically growing cells were diluted to 2.5 × 10^6^ cells/ml in fresh medium, and the density was measured hourly using a hemocytometer. To create dilutions for spot assays, logarithmically growing cells were diluted to 1.0 × 10^6^ cells/ml in phosphate-buffered saline (PBS). Serial dilutions of 10^5^, 10^4^, and 10^3^ cells/ml were made. Five microliters of cell culture dilutions was pipetted for each spot, and plates were incubated at 30°C for 2 days, unless otherwise indicated. To assess hyphal growth, cells were either plated onto Spider medium and incubated for 5 days at 30°C before imaging or were diluted 1:50 into fresh YPD medium supplemented with 10% fetal bovine serum (FBS) and incubated at 37°C to induce hyphae.

### Light microscopy.

Microscopy for static images was conducted using a Zeiss Axio Observer epifluorescence microscope with a 100× (1.40 NA) oil objective AxioCam hRM camera controlled by Axiovision software. Time-lapse imaging was conducted using the Olympus IX83 with a 100× oil objective (1.4 NA), Andor Zyla 4.2 Plus camera controlled by the cellSens software. For time-lapse and static imaging, logarithmically growing cells were immobilized between an agarose pad and a glass coverslip, as previously described (41). For time-lapses, images were captured in five z-slices 0.8 μm apart. Image stacks and pole-to-pole distances were analyzed with ImageJ (NIH). Graphs were calculated and displayed using GraphPad Software; figures were compiled in Adobe Photoshop and Adobe Illustrator.

### RNA sequencing.

Logarithmically growing yeast cells were harvested and centrifuged at 4,000 × *g* for 10 min at 4°C. Cell pellets were flash frozen in liquid nitrogen, and genomic DNA-free total RNA was extracted from each pellet by grinding the fungal mass to a fine powder and resuspending it in 1 ml TRIzol (Ambion) solution and using the RNeasy mini-spin columns (Qiagen) following the manufacturer’s protocol. RNA quantification was carried out spectrophotometrically at 260 nm and 280 nm, and RNA integrity was evaluated by NanoDrop2000 (Thermo Scientific). Total RNA (1 μg/sample) was shipped to the National Research Council of Canada, DNA Sequencing Technologies Facility (Saskatoon, Canada), where further quality check was performed using a BioAnalyzer followed by short cDNA fragment synthesis using the TruSeq Stranded RNALT kit, and finally sequenced on an Illumina HiSeq 2500 platform according to the manufacturer’s guidelines (Illumina, USA). The DESeq2-based SARTools (v1.5.1) pipeline as previously described (95) was adopted for differential analysis of mapped C. albicans Assembly 22 RNA-seq count data. A BH *P* value adjustment was performed (96, 97), and the false-discovery rate was set at *P* < 0.05.

### Data availability.

The RNA-seq data that support the findings of this study are provided in Table S1 and are available at the Sequence Read Archive (SRA) under BioProject accession no. PRJNA579546.
